# Effects of *Astragalus* polysaccharide supplementation on growth performance, carcass quality, muscle fiber characteristics, meat quality, and development of finishing pigs

**DOI:** 10.3389/fnut.2025.1654720

**Published:** 2025-08-08

**Authors:** Wenfeng Ma, Zhuo Ma, Xiaoli Zhang, Pei Mao, Mengmeng Gao, Lingping Zhao, Qiujue Wu

**Affiliations:** ^1^College of Animal Science and Technology, Henan University of Science and Technology, Luoyang, China; ^2^Veterinary Medicine and Engineering, Nanyang Vocational College of Agriculture, Nangyang, China

**Keywords:** *Astragalus* polysaccharides, growth performance, carcass quality, meat quality, muscle growth and development, finishing pig

## Abstract

This study systematically evaluated the effects of *Astragalus* polysaccharides (AP) on growth performance, carcass quality, muscle fiber characteristics, and meat quality in finishing pigs. A total of 80 crossbred pigs (86.30 ± 1.0 kg) of approximately 5 months of age were selected and randomly assigned four treatments (five replicates for each treatment and four pigs for each replicate) on the basis of their initial body weight. They were, respectively, fed a basal diet (control group), and a basal diet supplemented with 0.1, 0.2%, or 0.3% AP. The results showed that compared with the control treatment, supplementation with *Astragalus* polysaccharides significantly increased the final body weight (FBW), average daily gain (ADG), and average daily feed intake (ADFI), and improved the dressing percentage, loin eye area, lean meat percentage, pH_24h_, a*, cooking percentage, marbling scores, and intramuscular fat (IMF) of carcass and meat (*p* < 0.05). In addition, the AP treatments also increased the contents of phenylalanine, methionine, and several other amino acids in the meat; growth serum hormone (INS, IGFs, and T_3_) concentrations; muscle fiber cross-sectional areas; and the mRNA expression levels of *FBOX32* and *MyoG* in muscles (*p* < 0.05). Meanwhile, AP treatments also significantly decreased the feed-to-gain ratio (F/G), fat percentage, L*, b*, and shear force of meat, somatostatin (SS) and T_4_ serum concentrations, and muscle fiber diameters (*p* < 0.05). However, there were no significant differences in these parameters between the treatments with different levels of *Astragalus* polysaccharide supplementation these parameters (*p* > 0.05). In conclusion, this study demonstrates that the diet supplementation of *Astragalus* polysaccharides improves growth performance, carcass characteristics, and meat quality in finishing pigs. These improvements are evidenced by elevated altering amino acid compositions; optimized serum hormone concentrations related to growth; favorable muscle fiber characteristics; and upregulated the mRNA expression of key genes modulating muscle growth and development.

## Introduction

Pork is celebrated for its abundance of high-quality proteins, essential vitamins (such as vitamin B_12_), and various minerals, along with its superior palatability and distinct sensory appeal, including a fresh and aromatic flavor profile (1). Global demand for pork has grown significantly in recent decades, driven by escalating consumer preferences for nutritious and tasty meat products. However, the modern swine industry faces multiple challenges, such as high-density rearing, chronic stress, and excessive antibiotic use, which can adversely affect pig health and welfare—ultimately impairing pork quality. Specifically, pigs exposed to such stressors are more susceptible to muscle damage, leading to suboptimal meat color, compromised texture, and altered water-holding capacity (WHC), which are key indicators of meat quality deterioration (2).

To address these issues and enhance pork quality, the application of feed additives, especially herbal-derived feed additives, has attracted particular interest due to their abundance of bioactive compounds (e.g., flavonoids, alkaloids, and polysaccharides) that have beneficial effects on animal health and product quality (3). Lee et al. (4) indicate that herbal additives significantly improve the flavor of pork, enhance the intramuscular fat (marbling) content, and increase tenderness and juiciness, which are—traits highly valued by consumers. Xu et al. (5) reported that grape seed proanthocyanidin extract; enriche beneficial fatty acids (e.g., total polyunsaturated fatty acids (PUFAs) and n-3 PUFAs) in pork, thereby enhancing its nutritional value for human health, while also exerting positive effects on pig growth and immunity. Additionally, herbal additives have been shown to strengthen the immune system of pigs, reducing the incidence of diseases and decreasing reliance on antibiotics (6). As a result, they ultimately facilitate the production of higher-quality pork.

*Astragalus* membranaceus, a kind of traditional Chinese medicine, contains a variety of bioactive components, including polysaccharides, flavonoids, and triterpenoid saponins (6). These components possess various pharmacological effects such as cytoprotective, and antioxidative effects, antitumor activity, antiviral properties, anti-inflammatory activity, blood-glucose-reduction properties, and anti-aging effects, and are capable of bolstering the body’s immune function (7, 8). Numerous studies have demonstrated that *Astragalus* polysaccharides (AP) isolated from the roots of *Astragalus* membranaceus can augment the activities of antioxidant enzymes in serum and the liver; modulate gut microbiota composition; enhance immune function (7); promote the fat, glucose, and energy metabolism; enhancethe rumen fermentation in sheep (8); improve the growth performance and nutrient digestibility in piglets; strengthen the intestinal barrier function (3, 9); and enhance the meat quality of Furong crucial carps (10). Furthermore, one study found direct evidence that AP can treat muscle wasting, improve muscle atrophy, and increase the level of phosphorylated Akt (a hypertrophy-related protein) in skeletal muscles under insulin resistance (11). Although the effects of Ap—widely used in piglets—on muscles are notable, there is insufficient research on their application in finishing pigs, especially regarding their effects on carcass quality, muscle fiber characteristics, meat quality, and development. Hence, this study was designed to verify the probability that *Astragalus* polysaccharides may improve the carcass and meat quality, enhance meat amino acid contents, and growth-related serum hormones concentrations, and to underline the possible mechanisms behind this.

## Materials and methods

### *Astragalus* polysaccharides

The *Astragalus* polysaccharides (brown powder, purity > 98.0%) used in this experiment were obtained from Beijing Bai Ao Lai Bo Technology (Co., Ltd., Beijing, China).

The AP was uniformly mixed into the diet to ensure that each pig received the correct dose. The supplementation period lasted for 41 days.

### Animals, diets, and experimental design

A total of 80 healthy crossbred pigs (Duroc × Landrace × Large White) of approximately 5 months of age with an initial body weight of 86.30 ± 1.0 kg were randomly assigned to four dietary treatments based on sex. Each treatment group consisted of five replicates, with four pigs per replicate (half male and half female). The experimental treatments were as follows: the control treatment (pigs fed a basal diet), and the Astragalus polysaccharides treatments 1, 2, and 3 (pigs fed a basal diet supplemented with 0.1, 0.2, and 0.3% *Astragalus* polysaccharides (AP), respectively). The trial comprised a 3-day adaptation period followed by a 38-day experimental period.

The pigs were kept in a well-ventilated, temperature-controlled facility with 40 pens (2.2 m × 3.5 m each). Diets were formulated to meet the NRC (National Research Council) (12) standards for finishing pigs, as shown in Table 1. The pigs were fed twice daily at 7:00 and 17:00, and the pig house was regularly cleaned and disinfected to maintain hygiene and minimize disease risk. Fresh water was available ad libitum. The *Astragalus* polysaccharides were uniformly mixed into the diet to ensure that each pig received the correct dose. Meanwhile, the experimental design and procedures were approved by the Animal Care and Use Committee of Henan Science and Technology, adhering to China’s Regulations for the Administration of Affairs Concerning Experimental Animals.

**Table 1:** Composition and nutrient levels of the basal diet (air-dry basis), %.

Ingredients	Content (%)	Nutrient level	Content (%)
Corn	60.50	Calculated chemical composition	
Wheat	11.55	Digestible Energy(MJ/kg)^2^	13.88
Soybean meal	17.80	Crude Protein	14.89
DDGS	5.00	Ca	0.57
CaHPO_4_	1.20	Total Phosphorus	0.55
L-Lysine-HCl	0.30	Available Phosphorus	0.30
DL-Methionine	0.05	D-Lysine	0.88
L-Threonine	0.05	D-Methionine	0.35
Limestone	1.20	D-Met+D-Cys	0.52
NaCl	0.35	D-Threonine	0.50
Premix^1^	2.00	D-Tryptophan	0.15
Total	100.00		

1 Premix provided the following per kg of diets: Vitamin A 3000 IU; Vitamin D 250 IU; Vitamin E 18 IU; Vitamin K_3_ 0.5 mg; Vitamin B_1_ 0.50 mg; Vitamin B_2_ 5.00 mg; Vitamin B_5_ 12 mg; Vitamin B_6_ 2.00 mg; Vitamin B_12_ 0.02 mg; Folic acid, 0.3 mg; Nicotinic acid 20 mg; Pantothenic acid 14 mg; biotin 0.08 mg; Cu (CuSO_4_*·*5H_2_O) 100 mg; Fe (FeSO_4_·H_2_O) 75 mg; Mn 30 mg; Zn (ZnSO_4_·H_2_O) 45 mg.

2 DE was a calculated value, while the others were measured values.

### Data collection and sample processing

After a 24-h fasting period, the final live weights of the pigs were recorded on day 39. Blood samples (10 mL) were collected from each pig via jugular venipuncture and transferred into 1.5 mL microcentrifuge tubes. Subsequently, the samples were separated via centrifugation at 3,500 r/min for 15 min. After centrifugation, the serum from each sample was collected and stored at −80°C until analysis. After blood collection, eight pigs (two pigs/pen and four pens/treatment) were slaughtered at a commercial facility. Carcass weight was measured immediately post-slaughter. Samples of *longissimus dorsa* muscle (LDM) were immediately collected from the right side of the carcass; some were stored at 4°C for meat quality evaluation and others were frozen at −20°C for muscle chemical analysis. LDM samples for RNA extraction were treated with liquid nitrogen, and then stored at −80°C for further analysis. Fresh samples of LDM (1 cm^3^) were excised and placed into 4% paraformaldehyde in PBS (pH 7.3) for further morphological detection.

### Growth performance

At the end of the feeding trial, the body weights and feed intakes of each pig were recorded weekly. The average daily gain (ADG), average daily feed intake (ADFI), and feed-to-gain ratio (F/G) were calculated from these records. The calculation formulas used are as follows: ADG = (final body weight-initial body weight) / number of days in the experiment. The average daily feed intake (ADFI) was calculated by dividing the total feed intake of each group by the number of days and the number of pigs in the group. The F/G was calculated as ADFI / ADG.

### Analysis of carcass traits and meat quality

Before slaughter, the live weight of pigs was measured again. Pigs were electrically stunned and slaughtered by exsanguination. Subsequently, the carcasses were scalded, dehaired, and eviscerated in accordance with GB 12694–2016 (2016). After slaughter, the hot carcass weight of each pig was recorded and used to calculate the dressing weight and dressing percentage. Backfat thickness was measured using the first rib, last rib, and last lumbar vertebra at a three-quarters distance along the *longissimus dorsi* toward the belly. Muscle thickness at the penultimate 3 ~ 4 ribs was recorded and used to calculate the lean meat percentage. The loin eye area was measured at the level of the last rib. Other slaughter characteristic parameters, including bone weight, skin weight, fat weight, and plate oil weight, were measured and used to calculate other carcass parameters (such as: lean meat percentage and suet percentage).

The samples were defrosted, and the shear force of theLDM was measured with a WarnerBratzler shear device (SALTER®, G-R Elec. Mfg. Co. Collins Lane, MA). One meat sample (about 2 × 4 × 1 cm) was sliced parallel to the muscle fiber direction, weighed, and sheared at a right angle to the fibers using a 250 kg weight. The results are expressed in Newton (N). The meat color parameters of LDM samples (brightness, L*; redness, a*; and yellowness, b*) were measured 24 h after slaughter using a colorimeter (NRl0QC, 3nh, Shenzhen, China). The pH values of meat from the LDM at 45 min and 24 h post-slaughter were determined from the left-side carcass using a calibrated pH meter (pH-STAR, SFK-Technology, Denmark). Cooking loss was calculated by measuring the weight of muscle samples before and after cooking, starting 45 min post-slaughter. Drip loss was quantified as the percentage of weight change. The intramuscular fat (IMF) content was analyzed with a FoodScan Analyzer (Delta 320, Mettler-Toledo Group) by implementing methods described by Song et al. (13). The marbling scores were determined by a trained observer using published visual standards (14).

### Analysis of hydrolyzed amino acids in muscle

A total of 0.1 g of fresh muscle samples was placed into a container and, then 10 mL of 6 M hydrochloric acid was added. After ultrasonic vibration, the ampoule was sealed with a spray gun, placed in an oven, and the mixture was hydrolyzed at 110°C for 22 h. It was then removed and cooled down to 25°C. Then, a 0.3 mL aliquot of the solution was filtered through filter paper. The filtrate was collected in a quartz crucible and evaporated to dryness in a 70°C water bath. Subsequently, 3 mL of sample diluent was added. After hydrolysis, the hydrolyzed mixture was filtered to reach a constant volume through a 0.22-μm filter membrane; 1 mL of the resulting sample was analyzed using a high-speed amino acid analyzer (L-8900, Shimadzu, Japan).

### Growth-related serum hormones

The concentrations of growth hormone (GH), insulin (INS), insulin-like growth factors (IGFs), triiodothyronine (T_3_), somatostatin (SS), and thyroxine (T_4_) in serum were determined by using commercial kits and following the manufacturer’s instructions.

### Muscle fiber characteristics

To evaluate the characteristics of the muscle fiber cross-sectional area (CSA) and muscle fiber diameter, each muscle section was stained with H&E following the method described by Wang et al. (15). Briefly, muscle samples were fixed in 4% paraformaldehyde at room temperature for 24 h. Subsequently, they were embedded in paraffin and sectioned with a thickness of 5 microns using a cryostat (CM 1860, Leica Biosystems, Wetzlar, Germany). These sections were then stained with H&E. Representative areas were photographed under a Nikon 90i microscope (Nikon, Tokyo, Japan) at a magnification of 200×. An image analysis system (Image-Pro Plus 5.1; Media Cybernetics Inc., Rockville, MD) was employed to analyze the stained sections. For each muscle, three different points on three images, containing a total of approximately 300 muscle fibers without signs of tissue disruption or freeze damage, were selected for measurement. The fiber diameter (μm) and CSA (μm^2^) were then calculated.

### Quantitative reverse-transcription PCR analysis

Total RNA was extracted from samples using TRIZOL reagent (purchased from Beijing Solarbio Science & Technology Co., Ltd., Beijing, China). After determining the concentration and purity of the total RNA, cDNA was synthetized using a PrimeScriptTM RT reagent kit (Takara, Dalian, China). Subsequently, quantitative real-time PCR was performed by using a CFX connect real-time PCR detection system (CFX-96, Bio-Rad, Hercules, USA) and SYBR green mixture (Q711-02, Vazyme, Nanjing, China). The primer sequences are presented in Table 2.

**Table 2:** Primer sequence information.

Gene^1^	Primer sequence (5`-3`)	Length (bp)
*GAPDH*	F: ACTCACTTCTACCTTTGATGCT	100
	R: TGTTGCTGTAGCCAAATTCA	
*FBOX32*	AAGGGAACTCCTCCAGACC	104
	CCATCCGATACACCCAGAT	
*MyoG*	AAACTACCTGCCCGTCGACCTC	112
	GGTCCCCAGCCCCTTATCTTCC	
*MyoD*	CTATGATGACCCGTGTTTCG	101
	AGTGTTCCTCGGGCTTTAGG	
*Myf5*	GGATCAGCAACTCGGAAGC	126
	GCAGATGGTAGATGAGCCTGGAAC	
*MSTN*	GCACCAAGCAAAGCCCAGAGG	143
	AGCACCCACAGCGATCTACTACC	

1 GAPDH, glyceraldehyde-3-phosphate dehydrogenase; FBOX32, Muscle atrophy F-box protein 32 gene; MyoG, Myogenin gene; MyoD, Myogenic differentiation factor gene; Myf5, Myogenic factor 5 gene; MSTN, Myostatin gene.

In this study, *GAPDH* was selected as the endogenous control gene due to its stable expression across different experimental conditions. The PCR cycling conditions were as follows: an initial denaturation step at 95°C for 10 min to fully unwind the DNA strands, followed by 40 cycles of denaturation at 95°C for 15 s to separate the double-stranded DNA; annealing at 60°C for 60 s to allow the primers to bind to their complementary sequences; and extension at 72°C for 30 s to synthesize new DNA strands. After 40 cycles, a final extension step at 72°C was carried out for 30 s to ensure complete synthesis of all DNA fragments. The relative expression levels of target genes were calculated as the target gene to control gene (*GAPDH*) ratio according to the 2^−ΔΔCT^ method. This method normalizes the expression of target genes in the endogenous control and the calibrator sample, thereby enabling accurate comparison of gene expression levels among different experimental treatments.

### Statistical analysis

All experimental data were statistically analyzed using SPSS for Windows (version 26.0, SPSS Inc., Chicago, IL, USA). To assess differences among groups, a one-way analysis of variance (ANOVA) was initially performed. One-way ANOVA is a statistical technique used to determine whether there are any significant differences between the means of three or more independent (unrelated) groups. After the one-way ANOVA, Tukey’s multiple range tests were conducted. Tukey’s test is a post-hoc test that allows for pairwise comparisons between all groups in the ANOVA, thereby helping identify exactly which groups differ significantly from one another. The results of the statistical analyses was presented as the mean ± standard error of the mean (SEM). A significance level of *p* < 0.05 was set as the criterion for determining significant differences.

## Results

### Growth performance

The results regarding the growth performance of finishing pigs are shown in Table 3. The final body weight (FBW), ADG, and ADFI in the AP1, AP2, and AP3 treatment groupswere significantly higher (*p* < 0.05) compared to those in the control (CON) treatment group in finishing pigs. Conversely, the F/G was significantly lower in the treatment groups (*p* < 0.05), while there were no significant differences in FBW, ADG, ADFI, and F/G among the AP treatments (*p* > 0.05).

**Table 3:** Effects of *Astragalus* polysaccharides supplementation on growth performance of finishing pigs.

Items	Treatments				*p*-value
	CON	AP1	AP2	AP3	
IBW/kg	86.45 ± 0.25	86.40 ± 0.28	86.35 ± 0.39	86.29 ± 0.45	0.519
FBW/kg	113.20 ± 0.42^a^	121.26 ± 1.01^b^	123.05 ± 0.35^b^	120.63 ± 0.85^b^	0.016
ADG/g	703.95 ± 13.67^a^	917.37 ± 35.19^b^	965.79 ± 28.96^b^	903.68 ± 19.41^b^	0.024
ADFI/kg	2.43 ± 0.09^a^	2.74 ± 0.12^b^	2.79 ± 0.18^b^	2.73 ± 0.13^b^	0.028
F/G	3.45 ± 0.11^b^	2.99 ± 0.08^a^	2.89 ± 0.10^a^	3.02 ± 0.13^a^	0.005

^a-b^ Means within the same row that do not share a common superscript are significantly different (*P* < 0.05); *n* = 8.

### Carcass traits and meat quality

The results regarding the carcass traits and meat quality of finishing pigs are shown in Table 4. In comparison with the CON treatment, the dressing percentage, loin eye area, lean meat percentage, pH_24h_, a* value, cooking percentage, marbling scores, and IMF were significantly increased (*p* < 0.05) in the AP1, AP2, and AP3 treatment groups, while the fat percentage, L*, b*, and shear force were significantly decreased in the AP1, AP2, and AP3 treatment groups (*p* < 0.05). Nevertheless, no significant differences were observed in the dressing percentage, loin eye area, lean meat percentage, pH_24h_, a* value, cooking percentage, marbling scores, IMF, fat percentage, L*, b*, and shear force among the AP treatments (*p* > 0.05). Additionally, there were no significant differences in the pre-slaughter body weight, carcass weight, backfat thickness, suet percentage, pH_45min_, and drip loss between the CON and the AP treatments (*p* > 0.05).

**Table 4:** Effects of *Astragalus* polysaccharides supplementation on carcass traits and meat quality of finishing pigs.

Items	Treatments				*P*-value
	CON	AP1	AP2	AP3	
Body weight before slaughter/kg	113.51 ± 1.02	120.95 ± 3.20	125.05 ± 2.31	123.65 ± 3.61	0.153
Carcass weight/kg	87.84 ± 1.05	91.89 ± 1.68	94.88 ± 2.04	92.95 ± 1.27	0.084
Dressing percentage/%	74.72 ± 0.28^a^	75.54 ± 0.11^b^	75.92 ± 0.30^b^	75.61 ± 0.08^b^	0.028
Loin eye area/cm^2^	44.42 ± 1.63^a^	52.08 ± 1.96^b^	51.62 ± 2.30^b^	52.36 ± 2.04^b^	0.032
Backfat thickness/mm	21.20 ± 1.32	23.21 ± 2.05	22.85 ± 1.25	23.06 ± 2.01	0.311
Fat percentage/%	6.98 ± 0.62^b^	5.14 ± 0.60^a^	4.05 ± 0.52^a^	4.68 ± 0.49^a^	0.037
Lean meat percentage/%	62.13 ± 2.65^a^	68.92 ± 1.42^b^	70.02 ± 3.11^b^	69.51 ± 2.18^b^	0.041
Suet percentage/%	0.50 ± 0.08	0.45 ± 0.02	0.42 ± 0.03	0.43 ± 0.08	0.068
pH_45min_	6.41 ± 0.16	6.44 ± 0.10	6.55 ± 0.11	6.45 ± 0.07	0.395
pH_24h_	5.42 ± 0.05^a^	5.69 ± 0.07^b^	5.75 ± 0.03^b^	5.73 ± 0.03^b^	0.005
L*	40.44 ± 0.53^b^	34.69 ± 0.61^a^	34.05 ± 0.21^a^	34.83 ± 0.30^a^	0.012
a*	3.79 ± 0.20^a^	4.42 ± 0.17^b^	4.48 ± 0.11^b^	4.43 ± 0.17^b^	0.039
b*	4.91 ± 0.14^b^	4.56 ± 0.17^a^	4.40 ± 0.12^a^	4.60 ± 0.10^a^	0.034
Dropping loss/%	4.21 ± 0.17	3.76 ± 0.14	3.71 ± 0.13	3.78 ± 0.15	0.457
Cooking percentage/%	62.46 ± 1.45^a^	68.62 ± 2.26^b^	70.58 ± 3.11^b^	69.04 ± 1.57^b^	0.042
Shear force/N	48.25 ± 0.30^b^	41.60 ± 0.12^a^	40.43 ± 0.21^a^	41.62 ± 0.16^b^	0.022
Marbling scores	2.58 ± 0.16^a^	2.99 ± 0.11^b^	3.06 ± 0.13^b^	2.92 ± 0.17^b^	0.031
IMF/%	1.29 ± 0.25^a^	2.89 ± 0.31^b^	3.21 ± 0.19^b^	3.07 ± 0.40^b^	0.027

^a-b^ Means within the same row that do not share a common superscript are significantly different (*P* < 0.05); *n* = 8.

### Meat amino acid contents

The meat amino acid contents of finishing pigs are shown in Table 5. Compared with the CON treatment, phenylalanine, methionine, threonine, glutamic acid, histidine, aspartic acid, alanine, glycine, glutamine, serine, proline, arginine, asparagine, essential amino acids (EAAs), and total amino acids (total AAs) were significantly increased in finishing pigs after the AP1, AP2, and AP3 treatment of (*p* < 0.05). However, no significant differences were found in lysine, tryptophan, isoleucine, leucine, valine, and tyrosine contents among the CON and the AP treatments (*p* > 0.05).

**Table 5:** Effects of *Astragalus* polysaccharides supplementation on meat amino acid contents of finishing pigs (Fresh sample) g/kg.

Items	Treatments				*P*-value
	CON	AP1	AP2	AP3	
Lysine^1^	0.25 ± 0.02	0.30 ± 0.04	0.32 ± 0.07	0.35 ± 0.05	0.072
Tryptophan^1^	0.06 ± 0.01	0.06 ± 0.02	0.08 ± 0.01	0.07 ± 0.01	0.068
Phenylalanine^1*^	0.16 ± 0.01^a^	0.21 ± 0.02^b^	0.23 ± 0.03^b^	0.22 ± 0.01^b^	0.021
Methionine^1^	0.09 ± 0.02^a^	0.13 ± 0.03^b^	0.16 ± 0.01^b^	0.15 ± 0.02^b^	0.031
Threonine^1^	0.16 ± 0.01^a^	0.20 ± 0.03^b^	0.23 ± 0.02^b^	0.21 ± 0.01^b^	0.017
Glutamic acid^*^	0.13 ± 0.02^a^	0.23 ± 0.03^b^	0.28 ± 0.04^b^	0.26 ± 0.01^b^	0.025
Isoleucine^1^	0.16 ± 0.02	0.17 ± 0.01	0.19 ± 0.03	0.18 ± 0.01	0.059
Leucine^1^	0.27 ± 0.01	0.30 ± 0.01	0.35 ± 0.03	0.34 ± 0.02	0.084
Valine^1^	0.26 ± 0.02	0.29 ± 0.03	0.30 ± 0.04	0.28 ± 0.01	0.075
Histidine	0.06 ± 0.03^a^	0.09 ± 0.01^b^	0.10 ± 0.02^b^	0.08 ± 0.01^b^	0.018
Aspartic acid^*^	0.07 ± 0.01^a^	0.10 ± 0.02^b^	0.11 ± 0.01^b^	0.10 ± 0.03^b^	0.039
Alanine^*^	0.91 ± 0.02^a^	1.03 ± 0.05^b^	1.15 ± 0.07b	1.09 ± 0.06^b^	0.016
Glycine^*^	0.41 ± 0.02^a^	0.50 ± 0.03^b^	0.59 ± 0.01^b^	0.52 ± 0.02^b^	0.027
Glutamine	0.70 ± 0.10^a^	1.28 ± 0.09^b^	1.37 ± 0.04^b^	1.30 ± 0.08^b^	0.014
Serine	0.14 ± 0.01^a^	0.20 ± 0.02^b^	0.25 ± 0.01^b^	0.23 ± 0.03^b^	0.030
Proline	0.15 ± 0.02^a^	0.20 ± 0.03^b^	0.26 ± 0.01^b^	0.22 ± 0.02^b^	0.021
Arginine	0.16 ± 0.01^a^	0.23 ± 0.03^b^	0.26 ± 0.02^b^	0.24 ± 0.01^b^	0.011
Asparagine	0.07 ± 0.02^a^	0.10 ± 0.01^b^	0.12 ± 0.03^b^	0.11 ± 0.02^b^	0.015
Tyrosine^*^	0.19 ± 0.01	0.21 ± 0.02	0.22 ± 0.01	0.19 ± 0.03	0.063
EAA	1.41 ± 0.07^a^	1.66 ± 0.11^b^	1.86 ± 0.09^b^	1.80 ± 0.10^b^	0.028
Total AA	4.39 ± 0.20^a^	5.83 ± 0.15^b^	6.57 ± 0.23^c^	5.34 ± 0.17^b^	0.012

^a-b^ Means within the same row that do not share a common superscript are significantly different (*p* < 0.05); *n* = 8. * Is flavor amino acid.

1 is EAA (essential amion acid).

### Growth-related serum hormones

The growth-related serum hormones of finishing pigs are shown in Table 6. Compared to the CON treatment group, the concentrations of INS, IGFs, and T3 were significantly increased (*p* < 0.05), while the concentrations of SS and T_4_ were significantly decreased in finishing pigs after the AP1, AP2, and AP3 treatments of (*p* < 0.05). Furthermore, no significant difference was observed in the GH concentration among the CON and the AP treatments (*p* > 0.05).

**Table 6:** Effects of *Astragalus* polysaccharides supplementation on growth serum hormone of finishing pigs.

Items	Treatments				*P*-value
	CON	AP1	AP2	AP3	
GH/(ng/mL)	6.02 ± 0.13	6.32 ± 0.15	6.38 ± 0.34	6.34 ± 0.22	0.118
INS/(uIU/ML)	14.85 ± 0.13^a^	16.91 ± 0.30^b^	17.82 ± 0.21^b^	17.59 ± 0.25^b^	0.042
IGFs/(ng/mL)	161.96 ± 5.89^a^	205.38 ± 9.24^b^	226.35 ± 10.24^b^	219.67 ± 8.24^b^	0.024
SS/(pg/mL)	40.18 ± 1.12^b^	30.12 ± 3.04^a^	20.41 ± 1.07^a^	25.67 ± 2.11^a^	0.017
T3/(ng/mL)	0.51 ± 0.02^a^	0.67 ± 0.01^b^	0.71 ± 0.03^b^	0.70 ± 0.01^b^	0.023
T4/(ng/mL)	64.92 ± 5.01^b^	54.38 ± 2.35^a^	50.27 ± 1.38^a^	52.47 ± 3.04^a^	0.031

^a-b^ Means within the same row that do not share a common superscript are significantly different (*p* < 0.05); *n* = 8.

### Muscle fiber characteristics

The muscle fiber characteristics of finishing pigs are shown in Table 7. The muscle fiber cross-sectional area was significantly increased in finishing pigs after the AP1, AP2, and AP3 treatment compared to the CON treatment (*p* < 0.05). Moreover, the muscle fiber diameter was significantly decreased in finishing pigs after the AP1, AP2, and AP3 treatments compared to the CON treatment (*p* < 0.05). However, no significant differences were observed in muscle fiber cross-sectional areas and muscle fiber diameters among the AP treatments (*p* > 0.05).

**Table 7:** Effects of *Astragalus* polysaccharides supplementation on muscle fiber characteristics of finishing pigs.

Items	Treatments				*P*-value
	CON	AP1	AP2	AP3	
Muscle fiber diameter/μm	68.12 ± 1.28^b^	60.38 ± 1.34^a^	58.79 ± 1.69^a^	59.67 ± 2.01^a^	0. 019
Muscle fiber cross-sectional area/μm^2^	4125.35 ± 85.20^a^	5125.61 ± 115.02^b^	5398.67 ± 195.37^b^	5268.19 ± 185.34^b^	0.018

^a-b^ Means within the same row that do not share a common superscript are significantly different (*p* < 0.05); *n* = 8.

### Gene mRNA expression levels related to muscle growth and development

**Figure 1:**
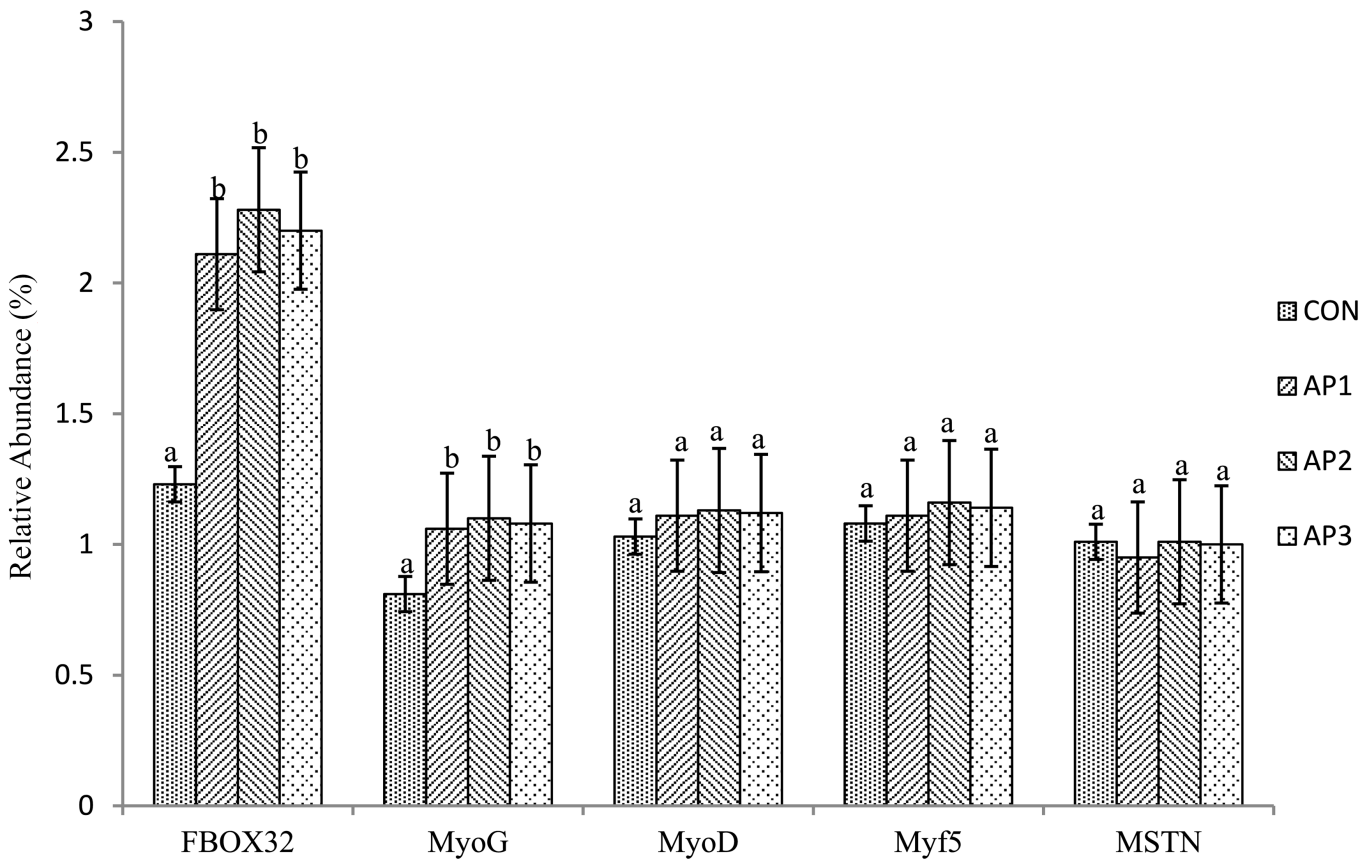
Effects of *Astragalus* Polysaccharides supplementation on the gene mRNA expression level related to muscle growth and development in the muscle of finishing pigs. ^a-b^ Means within the same row that do not share a common superscript are significantly different (*p* < 0.05); *n* = 8.

The gene mRNA expression levels related to muscle growth and development of finishing pigs are shown in Figure 1. The mRNA expression levels of *FBOX32* and *MyoG* in muscles were significantly increased in finishing pigs in the AP1, AP2, and AP3 treatment groups compared to the CON treatment group (*p* < 0.05). No significant differences were observed in *FBOX32* and *MyoG* mRNA expression levels in the muscle among the AP treatments (*p* > 0.05). Moreover, no significant differences were found between the *MyoD*, *Myf5*, and *MSTN* mRNA expression levels in muscles between the CON and the AP treatments (*p* > 0.05).

## Discussion

### Growth performance

*Astragalus* membranaceus has beneficial effects and has been used as feed additiveto increase ADG, and decrease the feed conversation ratio in animals (16–18). In the present study, pigs supplemented with *Astragalus* polysaccharides exhibited significant improvements in the ADG, ADFI, and F/G ratio, consistent with the findings of Wang et al. (9), who suggested that supplementation of polysaccharides led to a higher ADG and lower F/G in pigs. This phenomenon may be attributed to the following mechanisms: First, the enhancement of digestive enzyme (e.g., amylase and protease) and antioxidant enzyme (e.g., SOD and GSH-Px) activities, as well as the promotion of digestive juice secretion (17, 19, 20). Second, as key immunostimulants, polysaccharides likely modulate metabolic and physiological pathways to enhance animal health and growth performance (21). Third, these effects may correlate with the maintenance of intestinal morphology, promotion of intestinal epithelial development, and facilitation of beneficial bacterial colonization-critical factors for optimizing nutrient absorption and ultimately improving growth performance (22). Additionally, the results of this study indicate that this effect might be related to increased the concentrations of IGF-1, and enhanced the mRNA expressions of genes associated with growth (23).

### Carcass traits and meat quality

In this experiment, supplementing 0.1, 0.2%, or 0.3% AP in the diet significantly improved the dressing percentage, loin eye area, pH_24h_, and lean meat percentage in finishing pigs. While the supplementation of 0.1, 0.2%, or 0.3% AP improved the pre-slaughter body weight, carcass weight, backfat thickness, suet percentage, pH_45min_, and drip loss of finishing pigs to a certain extent, the effect was still not considered as significant. Similar results were also reported by Wang et al. (16), who reported that yellow broilers fed with a polysaccharide supplementation diet did not exhibit any significant differences in carcass quality, i.e., the weight and index of carcass, breast meat pH, and drip loss. Moreover, in the current study, dietary *Astragalus* polysaccharides also increased the cooking percentage, which is consistent with the findings of Wang et al. (16), who suggested that *Camellia oleifera Cake* polysaccharide supplementation increased the cooking loss and could improve the juiciness of meat.

The IMF content in pork is widely recognized as a critical determinant of meat quality and is directly associated with the marbling, juiciness, tenderness, and flavor of meat (24). In the current study, dietary AP significantly increased the IMF content and marbling scores in finishing pigs, indicating that AP have the potential to make improve tastier. The enhancement of pork flavor could be attributed to the improvements in the antioxidant abilities of pork and improvements to the gut microbiota composition (25). The results were consistent with a previous study, where *Pogostemon cablin* polysaccharides were used to increase IMP of broilers (26). Moreover, the enhancement of the meat flavor of finishing pigs meat might further lead to improved amino acid metabolism and amino acid levels, which is in accordance with the results of the present study.

The shear force values were also used to assess the tenderness of meat, which is another trait of importance for consumers and processors (27). Prior research has shown that supplementing of *Pleurotus citrinopileatus* polysaccharide in the diet is beneficial in terms of broiler meat tenderness (28), in agreement with our discoveries. In addition, this study indicated that *Astragalus* polysaccharides significantly reduced the muscle shear force of finishing pigs. Collectively, these findings indicated that *Astragalus* polysaccharide supplementation enhances the intramuscular lipid deposition and improves the juiciness of meat, thereby improving sensory attributes of pork muscle, such as the taste, tenderness, and flavor. This coule also be due to the fact that AP can improve the intestinal flora composition and increase the body’s immunity and antioxidant function (28).

The intensity of meat color is an important attribute for consumers who prefer pork with a higher pink intensity (29). In the present study, the meat color of finishing pigs fed a diet supplemented with *Astragalus* polysaccharides exhibited an increase in the a* value and a decrease in the L* and b* values. Similar results were reported by Sun et al. (30) and Zheng et al. (31) who noted that the supplementation of *Astragalus* polysaccharides could stabilize the meat color, increase the a* value, and reduce the b* value. This change in meat color might be related to the accumulation of oxymyoglobin. Sun et al. (30) confirmed that *Astragalus* polysaccharide could delay myoglobin oxidation and improve the meat color score. Previous studies have shown that antioxidants can improve some meat (pH, color, water-holding capacity, and tenderness) and eating (odor and tenderness) quality parameters, and also enhance the oxidative stability of pork (32, 33). Therefore, the effects of AP supplementation on meat quality may be related to its antioxidant properties. However, there is limited information available regarding the meat quality after the introduction of AP into the diet, and the mechanism by which AP affects meat quality remains unclear. Further studies are needed to comprehensively assess its effects on meat quality.

### Meat amino acid contents

Proteins are one of the most fundamental components of human and animal tissues, and are typically composed of various amino acids linked by peptide bonds. As amino acids are the building blocks of proteins, their composition, content, and balance directly determine the nutritional value of meat, while their profiles also significantly influence sensory attributes such as flavor and texture (34). Dietary factors are known to modulate amino acid availability and metabolism in animals, thereby impacting muscle amino acid deposition (34). The results of our present study show that supplementation of *Astragalus* polysaccharide can increase the contents of phenylalanine, methionine, glutamic acid, Thr, histidine, Asp., Ala, Gly, Gln, Ser, proline, Arg, asparagine, Tyr, EAA, and total AA in the *longissimus dorsi* muscle of finishing pigs. These results suggest that dietary AP improve the total amount of amino acids, the nutrition profile of meat, a muscle flavor, and growth performance of finishing pigs. These findings align with Chen et al. (35), who reported that AP supplementation in Tan sheep diets enhanced Gly, Asp., and TAA levels in the *longissimus dorsi* muscle, which might be closely related to the changes in gut flora induced by *Astragalus* polysaccharide addition (28).

Notably, supplementing different levels of *Astragalus* polysaccharide also increased the content of umami amino acids (UAAs) (including Ala, Gly, Gln, and Asp) and other flavor-related amino acids that are critical for muscle physicochemical properties and meat quality. Mechanistically, these effects may be attributed to AP-mediated improvements in digestive enzyme activity, antioxidant capacity, intestinal morphology, and gastrointestinal microbiota composition, all of which enhance protein and amino acid digestion, absorption, and utilization in finishing pigs (11). Additionally, AP may regulate the expression of amino acid metabolism-related genes (e.g., amino acid transporters or synthesizing enzymes) through its bioactive components, thereby influencing amino acid profiles in pork. However, the specific molecular pathways underlying these effects remain undefined and require further investigation. In conclusion, this study demonstrates that AP supplementation enhances the accumulation of flavor-related amino acids (e.g., Asp., Ala, Gly, and Gln) in pork, thereby improving meat quality. These findings highlight AP as a promising nutritional regulator for optimizing amino acid composition in livestock muscle tissues. Moreover, Xu et al. (5) also noted that the fatty acid composition of meat may influence both its nutritional quality and flavor. However, our current study lacks relevant data on meat fatty acids, which necessitates further analysis of fatty acid compositions and related mechanisms in future research.

### Growth-related serum hormones

The growth and development of animals are complex; are influenced by multiple factors such as environment, nutrition, age, and breed; and are regulated by various hormones in the body. Most hormones (such as INS, somatostatin, IGF-1, GH, T_3_, and T_4_) directly or indirectly play crucial roles in regulating body growth and development (36–38). These hormones collectively promote fat and protein accumulation, serving as reliable biomarkers for evaluating somatic growth and developmental status (23). Xu et al. (39) demonstrated that dietary supplementation with 1.0 ~ 2.0 g/kg dandelion polysaccharides significantly elevated serum INS levels, with 2.0 g/kg supplementation specifically enhancing IGF-1 concentrations. Consistent with this, previous studies have shown that dietary polysaccharide inclusion could upregulate serum IGF-1 secretion, increase insulin somatostatin concentration, and decrease somatostatins in animals (23, 40). Our study further validated these findings. Dietary supplementation with *Astragalus* polysaccharides increases IGF-1 and insulin concentrations, and decreases the somatostatin concentrations in the serum of finishing pigs. Collectively, these results suggest that the growth-promoting effects of plant polysaccharides may be mediated by regulating serum glucose levels and increasing glucose uptake and utilization in tissue cells, as well as increasing protein synthesis and glycogenesis (41), thereby influencing nutrient absorption and utilization and maintaining the normal health status of pigs.

Thyroid hormones T_3_ and T_4_, secreted by the thyroid gland and its peripheral tissues, play a pivotal role in regulating body energy intake, and metabolic processes (42). Zeng et al. (43) reported that *Astragalus* polysaccharide injection increased the serum T_3_ levels in dairy cows, likely by restoring metabolic function through elevated T_3_ concentrations. This finding is consistent with our current results on T_3_ levels in finishing pigs. Additionally, our study demonstrated that AP supplementation significantly increased the INS concentration in finishing pigs, suggesting that dietary AP may regulate the serum glucose levels by influencing insulin and glucagon secretion. Notably, we observed a significant decrease in T_4_ levels following AP treatment. This result implies that AP may promote the conversion of T_4_ to T_3_, by potentially accelerating tissue cell differentiation and maturation or by modulating the activity of 5′-deiodinase.

GH is one of the most important hormones in this regulatory network and is produced by other tissues through autocrine or paracrine mechanisms (44). After binding to its receptor, GH induces hepatocytes to produce IGF-1, which mediates tissue cell differentiation and maturation, enhances protein synthesis, and promotes the development of tissues and organs. Qiao et al. (45) reported that supplementation of *aloe* polysaccharides in piglet diets significantly increased GH levels and promoted growth, which is consistent with the results of our present study. In the present study, we found that AP caused no significant improvement in GH levels of finishing pigs, which contradicts the findings of Wu et al. (18), who suggested that AP increased GH serum concentrations in lactating cows. This discrepancy may be partly attributed to the change in cortisol release, which could in turn alter mRNA expression in the GH gene and the proliferation of muscle cells, as demonstrated in a previous study (40, 46). In conclusion, *Astragalus* polysaccharides may promote growth in finishing pigs by increasing the serum T_3_, INS, and IGF-1 levels, thereby regulating nutrient metabolism and tissue development.

### Muscle fiber characteristics

The present experiment demonstrated that *Astragalus* polysaccharide significantly increased muscle fiber cross-sectional areas and decreased muscle fiber diameters. This dual effect suggests enhanced muscle hypertrophy and structural optimization, which aligns with the proposed mechanism of action for this bioactive compound. However, t no reports have been identified regarding the impact of *Astragalus* polysaccharide on the muscle fiber cross-sectional areas and diameters in pork. However, related research on plant polysaccharides has demonstrated that *Glycyrrhiza* polysaccharide could increase the postmortem pH value of chicken breast meat while reducing its acidification, which is attributed to an increase in the cross-sectional area and a decrease in the diameter of muscle fibers (23). Additionally, studies have indicated that lower shear force and smaller fiber diameters are associated with superior sensory tenderness, water-holding capacity, flavor profile, and juiciness (24, 47, 48). These studies align with our results of meat quality, further verifying the positive effects of *Astragalus* polysaccharides on meat quality.

### Gene mRNA expression levels related to muscle growth and development

Polysaccharides, a diverse class of complex carbohydrates, have garnered increasing attention for their potential regulatory roles in cellular processes, particularly those mediating the muscle development, including genes such as *MyoD*, *MyoG*, *FBOX*, *MSTN*, and *Myf5*. Accumulating evidence highlights *MyoG* (myogenin), a key member of the myogenic regulatory factor (MRF) family, as a pivotal regulator of muscle fiber number and size and myoblast proliferation/differentiation, thereby shaping muscle growth and development (49–51). In the present study, dietary supplementation with AP significantly upregulated the *MyoG* mRNA expression, aligning with the results of Li et al. (23), who reported similar *MyoG* activation by glycyrrhiza polysaccharides. Notably, the AP-treated group exhibited improved meat quality parameters-including a reduced shear force, drip loss, and cooking loss-correlating with morphological changes in muscle fibers (e.g., increased diameter and cross-sectional area). These observations suggest that *MyoG* may directly influence both myofiber structural integrity and meat texture.

*MyoD* and *MSTN* (myostatin) genes are these genes are well-characterized regulators of muscle growth in animals (52, 53). While Deng et al. (54) demonstrated that *Chinese yam* polysaccharides significantly enhanced *MyoD* and *MSTN* expressions in broiler chickens, our study observed only a marginal, non-significant upregulation of these genes in finishing pigs supplemented with AP. This discrepancy may reflect species-specific responses or differences in polysaccharide dosage/type. Nevertheless, the trend toward *MyoD*/*MSTN* activation implies that optimized AP supplementation could potentially enhance myogenic programs in pigs, particularly when combined with nutritional strategies for amplifying anabolic signaling.

On the other hand, *Myf5*, a member of the *MyoD* family of basic helix–loop–helix (bHLH) transcription factors, serves as one of the earliest markers of the myogenic lineage and is indispensable for committing mesenchymal stem cells to muscle cell fate (55). Its expression initiates the myogenic program, thereby driving the specification of myoblasts of muscle progenitor cells. In the context of muscle physiology, certain *FBOX* proteins act as key regulators of myogenic fate by targeting myogenic regulatory factors (MRFs) for ubiquitin-proteasome-mediated degradation. Recent studies have investigated the impact of polysaccharides on *FBOX* and Myf5 expressions. Natural-source polysaccharides, such as those from plants and fungi, have been shown to modulate myogenic pathways. Zhao et al. (56) reported that polysaccharides from a marine alga upregulated the *Myf5* expression in myoblast cell lines, with proposed mechanisms involving activation of the mitogen-activated protein kinase (MAPK) pathway. Specifically, these polysaccharides may bind to cell surface receptors, triggering phosphorylation cascades (e.g., ERK1/2 and p38 MAPK) that enhance *Myf5* transcription. In our study, dietary supplementation with *Astragalus* polysaccharides (AP) tended to increase *Myf5* mRNA expression in finishing pigs, albeit not significantly, which may reflect dosage-dependent effects or species-specific responses.

Regarding *FBOX* proteins, the role of polysaccharides in muscle homeostasis is bidirectional. While some bioactive polysaccharides (e.g., in catabolic states) downregulated the atrophy-related *FBOX32* mRNA expression via inhibiting glucocorticoid or NF-κB pathways (8), our study observed a marginal upregulation of *FBOX32* mRNA expression levels in AP-supplemented pigs. This apparent contradiction may stem from differences in experimental models (e.g., stress-induced atrophy vs. normal growth conditions) or polysaccharide subtypes. Notably, the concurrent upregulation of *MyoG* and *Myf5* mRNA expression levels in our study suggests that AP may prioritize myogenic differentiation over atrophy pathways, potentially by enhancing MRF activity despite the modest *FBOX32* induction. Collectively, these findings indicate that AP promotes muscle development in finishing pigs by modulating the expression of key myogenic genes (*MyoG*, *MyoD*, *MSTN*, *Myf5*, and *FBOX*). The observed effects on key myogenic genes likely underlie AP’s ability to improve nutrient absorption, promote myofiber formation, and alter the fiber composition; however, the molecular mechanisms (such as MAPK pathway activation or receptor-mediated signaling) require further validation.

## Conclusion

Dietary supplementation with *Astragalus* polysaccharides has been shown to improve growth performance and carcass traits in finishing pigs. Specifically, AP significantly modulate amino acid composition and meat quality parameters; these improvements are likely associated with the regulation of growth-related serum hormones, muscle fiber characteristics, and gene mRNA expression levels involved in muscle growth and development. These findings suggest that AP could be used as a promising natural feed additive for enhancing growth performance and meat quality of finishing pigs, thereby serving as a sustainable alternative to synthetic growth promoters. Future research should dissect the underlying pathways in order to clarify how polysaccharides balance muscle anabolism and catabolism.

## Data Availability

The raw data supporting the conclusions of this article will be made available by the authors, without undue reservation.
